# ddPCR Enhances early diagnosis, treatment, prognosis, and pathogen verification in elderly BSI

**DOI:** 10.3389/fcimb.2025.1605795

**Published:** 2025-07-10

**Authors:** Jiayi Peng, Huili Bai, Ying Li, Huating Luo, Jiajun Li, Haifeng Dai, Hongmei Wang, Tao Meng, Jia Zhang, Zhijian Wang, Xuanxin Chen, Wei Cheng, Yan Peng, Wenxiang Huang

**Affiliations:** ^1^ Department of Geriatrics, The First Affiliated Hospital of Chongqing Medical University, Chongqing, China; ^2^ The Center for Clinical Molecular Detection, The First Affiliated Hospital of Chongqing Medical University, Chongqing, China; ^3^ Department of Infection, The First Affiliated Hospital of Chongqing Medical University, Chongqing, China; ^4^ Department of Nursing, The First Affiliated Hospital of Chongqing Medical University, Chongqing, China; ^5^ Department of Surgical Intensive Care Unit, The First Affiliated Hospital of Chongqing Medical University, Chongqing, China

**Keywords:** BSI, diagnosis, prognosis, ddPCR, elderly, false positive

## Abstract

**Background:**

Bloodstream infection (BSI) exhibits elevated mortality, particularly among elderly patients manifesting atypical symptoms. Although blood culture (BC) remains the diagnostic gold standard, its limited sensitivity and prolonged turnaround time impede early detection. Droplet digital polymerase chain reaction (ddPCR), a novel pathogen detection method with superior sensitivity and rapid results, demonstrates significant diagnostic and prognostic for BSI. However, heightened sensitivity may increase false positive rates, with elderly patients particularly susceptible to specimen contamination and transient bacteremia.

**Methods:**

This retrospective study employed clinical judgment as the diagnostic reference. Patients were stratified into BSI and non-BSI groups, with data collected on ddPCR and BC results, imaging and laboratory findings, medication response, and discharge outcomes. The diagnostic accuracy and antibiotic guidance efficacy of ddPCR and BC were compared, and the clinical utility of ddPCR was evaluated for prognostic assessment and false positive identification.

**Results:**

The analysis encompassed 355 episodes from 280 elderly patients with suspected BSI. ddPCR demonstrated significantly higher detection rates compared to BC in BSI group (59.33% versus 20.57%). Combined implementation increased detection to 65.07%. Regardless of clinical judgment (59.61% versus 20.57%) or alternative microbiological tests (90.63% versus 7.14%) served as the reference standards, ddPCR exhibited superior sensitivity to BC. No significant differences emerged in antibiotic adjustment rates or therapeutic efficacy between ddPCR and BC. Elevated microbial species diversity correlated with unfavorable discharge outcomes (P<0.001, OR=2.122). Multiple follow-up ddPCR monitoring revealed progressive increases in the number of species and the copies of some (or all) species among patients with poor outcomes, contrasting with decreasing trends in those with favorable outcomes. When detecting *Streptococcus*, coagulase-negative *Staphylococci* (CoNS), *Acinetobacter baumannii* complex, and *Candida*, diagnostic thresholds of 132.55, 182.70/262.24, and 174.78 copies/mL, respectively, were established to help differentiate false-positive results.

**Conclusion:**

The combination of ddPCR with BC improves BSI diagnosis in elderly patients and facilitates antibiotic treatment optimization. Moreover, ddPCR demonstrates potential for prognostic evaluation and false-positive discrimination. Nevertheless, these findings require further validation through large-scale prospective studies employing predefined clinical criteria.

## 1 Introduction

Bloodstream infection (BSI) and its progression to sepsis and septic shock represent major causes of global mortality (Rudd et al., 2020). Substantial evidence indicates that delayed or inappropriate initial antibiotic administration significantly reduces both short-term and long-term survival in sepsis patients (Peltan et al., 2019; Kollef et al., 2021). Elderly individuals face increased risk of missed or incorrect BSI diagnosis due to atypical clinical manifestations and subtle alterations in infection markers (Hyernard et al., 2019; Laupland et al., 2021; Leibovici-Weissman et al., 2021).

Current guidelines (Evans et al., 2021) advocate prompt initiation of broad-spectrum antibiotics for patients with high suspicion of sepsis or septic shock to enhance clinical outcomes. Nevertheless, extended broad-spectrum antibiotics use may elevate adverse event risks (Ong et al., 2017; Tamma et al., 2017; Baggs et al., 2018; Branch-Elliman et al., 2019), particularly among geriatric populations (Leibovici-Weissman et al., 2021). Consequently, rapid and accurate etiological diagnosis of BSI is critical for antibiotic regimen optimization and outcome improvement in elderly BSI patients.

Blood culture (BC) remains the diagnostic gold standard for bacterial and fungal BSI. However, this method suffers from prolonged turnaround times (Ehren et al., 2020; Kim et al., 2021). In early-stage of BSI, BC sensitivity decreases significantly due to low microbial concentrations in circulation and prior antibiotic administration (Pilecky et al., 2019). Emerging molecular detection technologies, including meta-genomic second-generation sequencing (mNGS), real-time quantitative polymerase chain reaction (RT-qPCR) and droplet digital polymerase chain reaction (ddPCR), aim to overcome these limitations (Tjandra et al., 2022). As the third-generation PCR technology succeeding RT-qPCR (Chen et al., 2021), ddPCR offers several advantages: reference curve independence, minimal blood PCR inhibitors interference, high sensitivity (≤10 CFU/mL), rapid detection speed (TAT ≤ 1 hour), and cost-effectiveness (Abram et al., 2020; Merino et al., 2022). Given these characteristics, ddPCR demonstrates utility not only for early BSI diagnosis but also for monitoring pathogen load dynamics to evaluate disease progression and prognosis. Unlike mNGS, ddPCR remains limited by fundamental PCR principles, restricting detection of uncommon and unidentified pathogens. Nevertheless, within its detectable spectrum, ddPCR offers superior speed and sensitivity compared to conventional methods (Hu et al., 2021).

Microorganisms including *Acinetobacter baumannii* (*A. baumannii*), *Streptococci*, coagulase-negative *Staphylococci* (CoNS) and *Candida* species frequently yield positive test results due to non-infectious factors such as contamination or transient bacteremia/fungemia, despite their pathogenic potential in immunocompromised and hospitalized populations (Fournier and Richet, 2006; Mea et al., 2021; Knauf et al., 2022; Lopes and Lionakis, 2022). Elderly patients exhibit increased susceptibility to false-positive results (transient bacteraemia and specimen contamination) attributable to extended healthcare facility exposure, compromised vascular integrity, and diminished skin barrier function. While BC benefits from standardized interpretation protocols developed through extensive clinical application (Centers For Disease Control And Prevention), ddPCR as an emerging technology currently lacks comparable established criteria.

Clinical trials have validated the diagnostic efficacy, accuracy, and sensitivity of ddPCR for etiological identification (Hu et al., 2021; Wu et al., 2022; Lin et al., 2023), along with its prognostic predictive value (Ziegler et al., 2019; Shao et al., 2022). Existing investigations, however, have predominantly concentrated on adult patients, with scarce data available for elderly patients. Moreover, prior studies failed to examine the diagnostic impact of false-positive findings.

A single-center retrospective study was conducted to compare the diagnostic efficacy and antibiotic guidance capability between BC and ddPCR, assess the prognostic value of ddPCR, and determine the threshold for distinguishing non-pathogenic microbiota detected by ddPCR. The analytical spectrum of ddPCR in this investigation encompassed *Pseudomonas aeruginosa*, *Enterobacter cloacae*, *Klebsiella* species, *Escherichia coli*, *A. baumannii*, *Staphylococcus* aureus, CoNS, *Enterococcus* species, *Streptococcus* species and *Candida* species.

## 2 Methods

### 2.1 Study population and data collection

**Figure 1:**
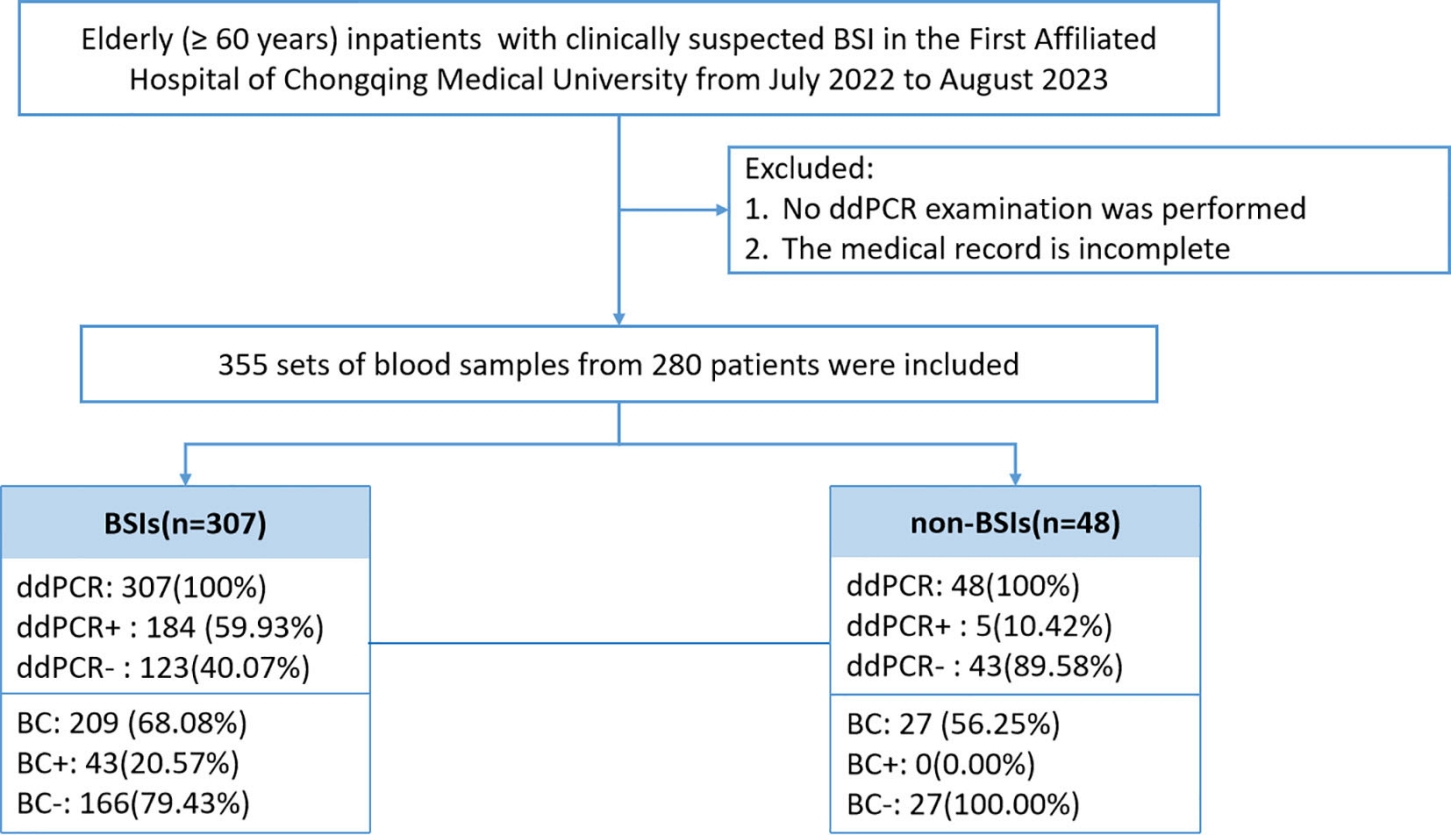
Patient enrollment flow chart and result analysis.

This investigation included elderly patients (≥60 years) admitted to the First Affiliated Hospital of Chongqing Medical University with suspected BSI between July 2022 and August 2023 (**Figure 1**). Exclusion criteria comprised patients with incomplete medical records or those who did not undergo ddPCR testing. Detailed inclusion and exclusion criteria are provided in **Supplementary Table 1**. Medical records and laboratory results were obtained from the electronic medical record system of the First Affiliated Hospital of Chongqing Medical University.

### 2.2 Plasma deoxyribonucleic Acid extraction and ddPCR testing

Peripheral venous blood samples (5-10 mL) collected in EDTA anticoagulation tubes underwent centrifuged at 1200 × g for 5 minutes at 4°C to separate the upper plasma for subsequent nucleic acid extraction. Plasma nucleic acids were isolated using the Easy-CF2 extraction reagent (Pilot Gene Technologies, China) with the Auto-Pure nucleic acid purification system (Model 10B, Hangzhou Allsheng Instruments, China), processing 2 mL plasma supernatant in approximately 30 minutes according to manufacturer protocols. The reaction solution was transferred into individual inlet cups of the digital PCR microdroplet chip channels, followed by droplet generation using the DG32 system (Piloting Gene Technology, China). Microdroplet-containing chips were transferred to the TC1 PCR amplification system (Piloting Gene Technology, China) for thermal cycling under the following conditions: initial denaturation at 95°C for 5 mins; 40 cycles of denaturation at 95°C for 15 s and annealing/extension at 60°C for 30 s; followed by final cooling to 25°C for 1 min. Following PCR, each chip was loaded into the CS7 biochip analyzer (Piloting Gene Technology, China) for fluorescence scanning and data analysis through six detection channels (**Supplementary Table 2**). Result interpretation was performed in accordance with the manufacturer protocols. The analytical sensitivity threshold was established at 45 copies/mL.

### 2.3 Definitions

**Diagnosis of BSI**: The diagnosis of BSI was established through clinical evaluation. Two independent infectious disease specialists classified enrolled patients as BSI or non-BSI based on comprehensive assessment of clinical manifestations, laboratory findings, imaging examinations, therapeutic response, disease outcomes, and criteria outlined in the *NHSN Patient Safety Component Manual (* Centers For Disease Control And Prevention). Cases with diagnostic discrepancies underwent adjudication by a third specialist.

**Improvement after treatment**: Antimicrobial therapy response was defined as sustained reduction for three consecutive days in at least two of the following parameters: maximum body temperature, white blood cell count (WBC), C-reactive protein level (CRP), or procalcitonin concentration (PCT).

**Test turnaround time (TAT)**: TAT was defined as the interval between specimen collection and result reporting. BC analysis incorporated three reporting stages: (1) Initial positive alert reporting Gram stain findings and microbial morphology; (2) Subsequent organism identification results; (3) Final antimicrobial susceptibility testing (AST) results. For study purposes, BC TAT calculation utilized the first reporting point as the endpoint.

**Classification of antibiotics**: Antibiotic classification was determined through multidisciplinary team consensus with reference to established methodology (Moehring et al., 2021) (detail in **Supplementary Table 3**). Treatment medications involving conversion from oral to intravenous administration, irrespective of antibiotic class, were categorized as escalation. Conversely, intravenous-to-oral transitions represented de-escalation.

**Clinical Outcome Classification**: Patient discharge outcomes were categorized as positive outcomes including discharge with documented clinical improvement or stable condition and negative outcomes comprising in-hospital mortality or discharge against medical advice.

**Determination of false-positive microorganism**: Positive ddPCR results for *Streptococcus*, CoNS, *A. baumannii* complex, and *Candida* species underwent independent evaluation by two infectious disease specialists to identify potential false positives resulting from contamination or transient bacteremia. Assessment criteria included: patient-specific risk factors, pathogen findings from other sites, antibiotic efficacy, and clinical outcomes. In case of disagreement, a third infectious disease specialist was consulted to reach a consensus.

### 2.4 Data analysis

Continuous variables with normal distribution were expressed using mean, maximum, and minimum values, while median and interquartile ranges were employed otherwise. for non-normally distributed data. Categorical variables were analyzed using chi-square tests. For continuous variables, independent t-tests were applied to normally distributed data and Mann-Whitney U tests to non-normally distributed data. Statistical significance was defined as P < 0.05. All statistical analyses and graphical representations were performed using R software (version 4.4.2; R Foundation for Statistical Computing), SPSS (version 27.0; IBM) and, Microsoft Office applications.

## 3 Results

### 3.1 Clinical characteristics of patients

The analysis included 280 elderly patients with suspected BSI were included, from whom 355 blood sample sets were collected. ddPCR and BC testing within 24 hours was performed on 236 samples (**Figure 1**). Based on clinical assessment, participants were categorized into BSI and non-BSI groups. Demographic characteristics revealed no significant differences in median age, gender ratio, and comorbidities. However, the BSI group demonstrated markedly elevated inflammatory markers compared to non-BSI patients: PCT (1.32 ng/mL versus 0.22 ng/mL, p<0.001) and CRP (102.5 ng/mL versus 29.1 ng/mL, p<0.001). WBC showed no significant variation (P=0.118). Clinical outcomes analysis indicated superior discharge rates with documented improvement among non-BSI patients discharged with improvement was significantly higher (76.74% versus 42.19%, p<0.001), while hospitalization duration showed no statistically significant difference among groups (**Table 1**).

**Table 1:** Baseline characteristics of patients in the two groups.

Characteristic	Total	BSI Group	Non-BSI group	P values
Number of tests	280	237	43	
Age, median (IQR)	72.5 (67.00-80.75)	72 (67.00-80.50)	75 (67.00-80.50)	0.680
Male, n (%)	193 (68.9%)	162 (68.4%)	31 (72.1%)	0.626
Comorbidities, n (%)				
Cardiovascular disease	146 (52.14%)	120 (50.63%)	26 (60.47%)	0.235
Diabetes	68 (24.29%)	62 (26.16%)	6 (13.95%)	0.085
Malignancy	99 (35.36%)	81 (34.18%)	18 (41.86%)	0.332
Chronic lung disease	40 (14.29%)	36 (15.19%)	4 (9.30%)	0.310
Other diseases	44 (15.71%)	35 (14.77%)	9 (20.93%)	0.307
Laboratory examination,median (IQR)				
WBC (*10^9)	9.35 (5.69-14.11)	9.88 (5.60-12.01)	8.22 (5.69-12.01)	0.118
CRP (mg/L)	90.80 (168.00-32.00)	102.50 (42.93-183.75)	29.1 (10.1-41.1)	<0.001
PCT (ng/mL)	0.72 (0.21-5.26)	1.32 (0.24-6.46)	0.22 (0.16-0.31)	<0.001
Discharge Outcomes				
Discharged with improvement,n (%)	133 (47.5%)	100 (42.19%)	33 (76.74%)	<0.001
Length of stay (d), median (IQR)	18.5 (10.00-31.00)	18 (9.50-31.00)	19 (11.00-29.00)	0.822

IQR, interquartile range; d, days; WBC, white blood cell; CRP, C-reactive protein; PCT, procalcitonin.

### 3.2 Comparison of diagnostic efficacy of ddPCR and BC

(1) Microbiological detection performance analysis

**Figure 2:**
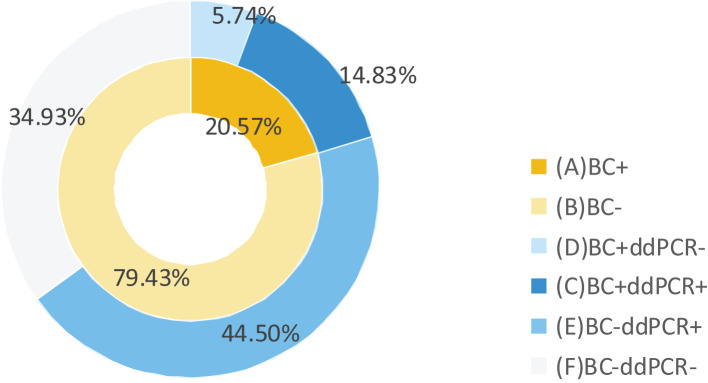
Comparison of ddPCR and BC detection results in BSI patients. BC+, positive results of BC; BC-, negative results of BC; ddPCR+, positive results of ddPCR; ddPCR-, negative results of ddPCR.

Among 377 documented BSI episodes, 209 cases underwent simultaneous ddPCR and BC testing within 24 hours. The positive rates were 59.33% positivity versus 20.57% for BC, with combined methodology achieving 65% detection efficiency (**Figure 2**).

**Figure 3:**
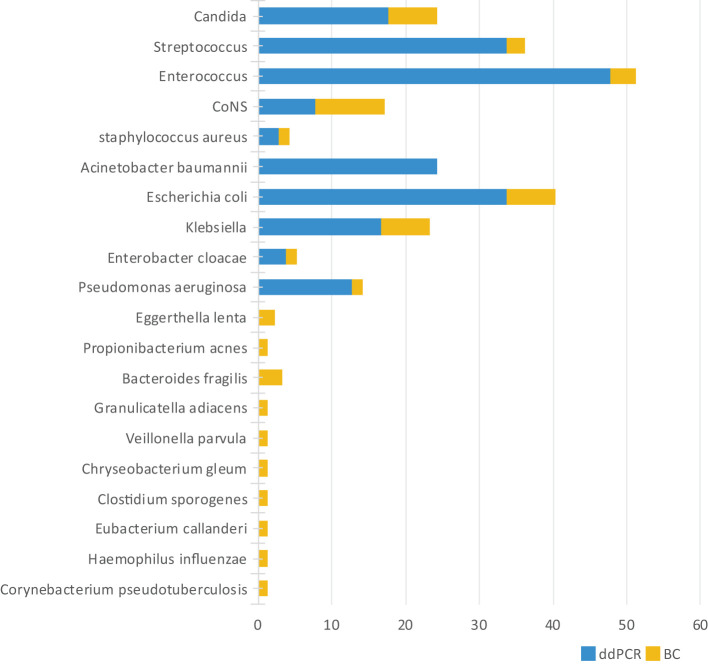
Distribution of species (genera) detected by ddPCR and BC.

Microbiological profiling identified 251 distinct bacterial species/genera, with ddPCR detecting 203 (80.88%) compared to 48 by BC (19.12%). The ddPCR platform captured 94.82% (238/251) of all identified microorganisms. The 5.18% (13/251) of species/genera out of the ddPCR detection range included *Corynebacterium pseudotuberculosis* and *Haemophilus influenzae*, among others. Notably, with the exception of *Haemophilus influenzae* and *Bacteroides fragilis*, these ddPCR-negative organisms generally represent non-pathogenic flora in clinical contexts (**Figure 3**).

**Figure 4:**
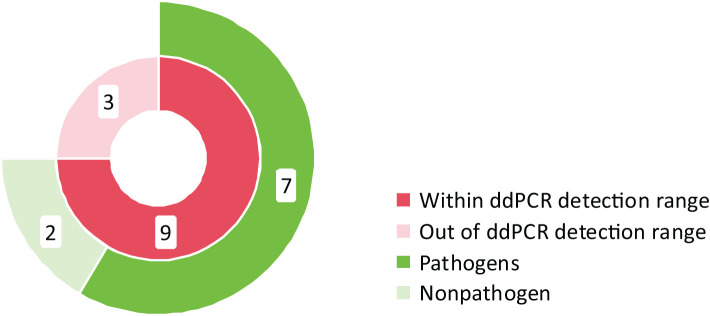
Analysis of BC+ and ddPCR- results.

Twelve specimens exhibited positive BC but negative ddPCR (**Figure 4**). Among these discordant results, nine cases involved microorganisms within the ddPCR detection range, including two considered contaminants or transient bacteremia (*Staphylococcus hominis*). The remaining seven cases comprised clinically significant pathogens: *Staphylococcus epidermidis* (n=2), *Klebsiella pneumoniae* (n=2), *Staphylococcus aureus* (n=1), *Staphylococcus haemolyticus* (n=1), and *Staphylococcus capitis* (n=1). Three specimens contained organisms outside the ddPCR detection range, specifically *Corynebacterium aurimucosum* (n=1), *Streptococcus gallolyticus* subsp. pasteurianus (n=1), and *Haemophilus influenzae* (n=1).

(2) Comparison of diagnostic performance metrics between ddPCR and BC

**Figure 5:**
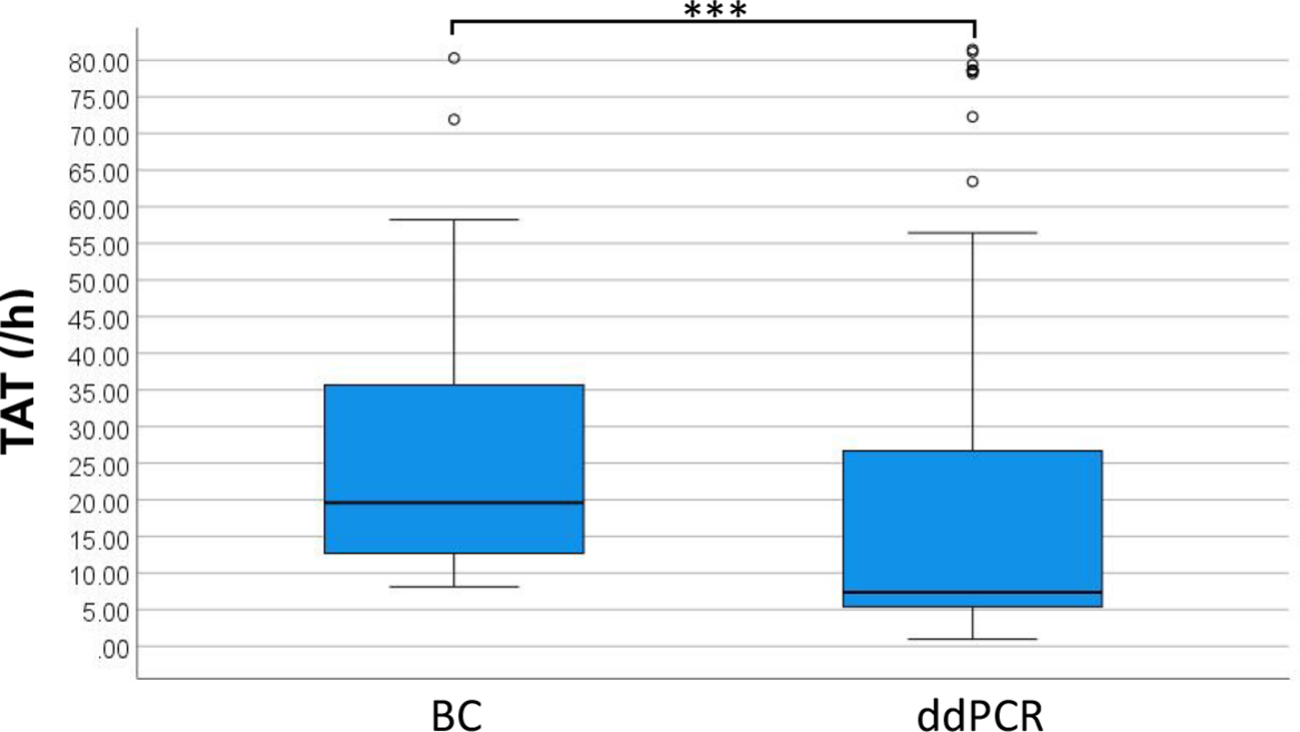
Distribution of TAT for ddPCR and BC positive results. ***P<0.001.

This study evaluated 355 cases of ddPCR and 236 cases of BC test results to compare diagnostic performance metrics including sensitivity, specificity, negative predictive value (NPV) and positive predictive value (PPV) (**Table 2**). The analysis demonstrated significantly superior sensitivity for ddPCR compared to BC, while specificity, PPV, and NPV showed no statistically significant differences between methodologies. From the total cohort, 109 blood sample sets met stringent inclusion criteria, requiring concurrent ddPCR and BC results within 24 hours plus supplementary etiological confirmation within 7 days through either alternative site testing or next-generation sequencing (mNGS/tNGS) of blood samples. When benchmarked against these comprehensive etiological references, ddPCR achieved 90.63% sensitivity, with PPV values significantly exceeding those obtained by conventional BC methodology (**Table 2**). For positive results, the median TAT for ddPCR was 7.38 hours, compared to 19.60 hours for BC, excluding strain identification. Negative BC results uniformly required 5 days for reporting. The distribution of TAT for positive results between the two methods is presented in **Figure 5**, demonstrating a statistically significant difference (p < 0.001).

**Table 2:** Comparison of diagnostic performance of ddPCR and BC.

Specimen Type	Detection method	Detection result	Clinical Assessment		Sensitivity (%) (95% CI)	Specificity (%) (95% CI)	PPV (%) (95% CI)	NPV (%) (95% CI)
			Positive	Negative				
Total Samples	ddPCR	Positive	183	5	59.61 (53.87-65.10)	89.58 (76.56-96.10)	97.34 (93.57-99.02)	25.75 (19.44-33.19)
		Negative	124	43				
	BC	Positive	43	0	20.57 (15.44-26.82)	100.00 (84.50-100.00)	100.00 (89.79-100.00)	13.99 (9.58-19.88)
		Negative	166	27				
Discordant samples	ddPCR	Positive	58	39	90.63 (80.05-96.13)	13.33 (5.54-27.48)	59.79 (49.33-69.47)	50.00 (22.29-77.71)
		Negative	6	6				
	BC	Positive	5	20	7.14 (2.66-16.57)	33.33 (17.94-52.86)	20.00 (7.61-41.30)	13.33 (6.92-23.61)
		Negative	65	19				

(3) The relationship between microbial loads and BC results

**Figure 6:**
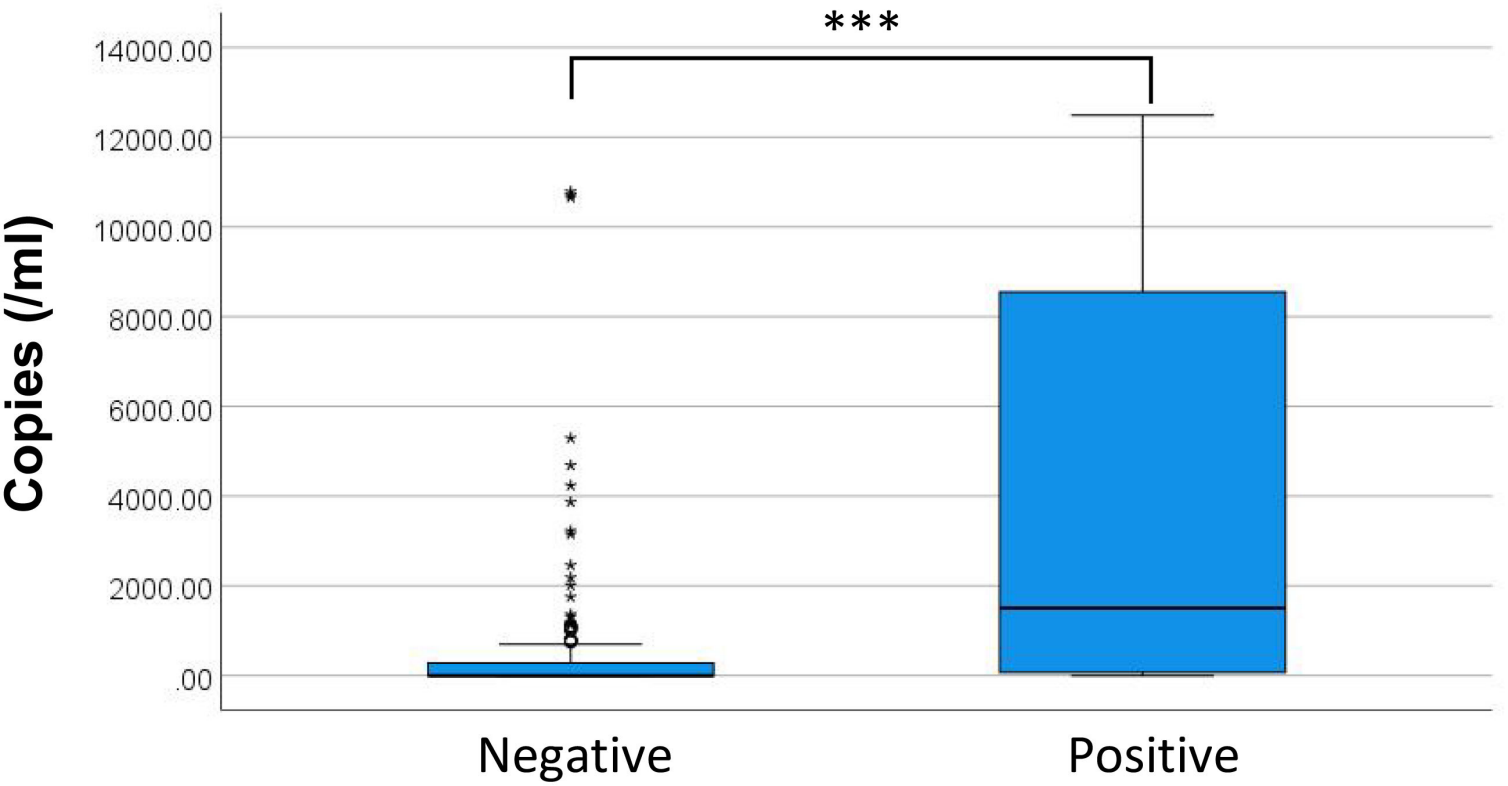
Maximum copies distribution for different BC results. ***P<0.001. * discrete values.

**Figure 6** presents the distribution of DNA copy numbers between BC+ and BC- groups. The P50 (P25, P75) DNA copies for BC- and BC+ were 0.00 (0.00, 282.35) copies/mL and 73.11 (1508.26, 8551.13) copies/mL, respectively. Spearman correlation revealed a positive correlation between maximum DNA copies and BC+ status (P < 0.05, r_s_ = 0.333). Although binary logistic regression was initially considered, the Hosmer-Lemeshow test rejected model fit (P = 0.007), suggesting the relationship between microbial DNA load and culture positivity does not follow a logistic pattern. discrepancy likely reflects differential microbial load thresholds required for culture positivity across various bacterial species/genera.

### 3.3 Comparison of the antibiotic stewardship efficiency between ddPCR and BC

(1) Antibiotic adjustment guided by ddPCR and BC

Among BSI group, ddPCR detected 183 cases positive results,. Among these, with subsequent antibiotic regimen modifications implemented in 45 cases (24.59%). Concurrently, BC identified 43 positive cases, among which 9 cases (20.93%) received antibiotic adjustments. The detailed antibiotic adjustment strategies are presented in **Table 6**.

(2) The effectiveness of antibiotic adjustment guided by ddPCR and BC

To evaluate the clinical effectiveness of antibiotic adjustment guided by ddPCR and BC, patients with ddPCR+ or BC+ (or both) who continued treatment were analyzed. Among 158 ddPCR+ cases, clinical improvement occurred in 95/158 (60.13%) when antibiotic therapy correlated with pathogen detection results. Similarly, 16/30 BC+ cases (53.33%) demonstrated improvement following pathogen-directed antibiotic adjustment. Comparative analysis revealed no statistically significant difference in treatment efficacy between the two diagnostic approaches (**Table 3**).

**Table 3:** Comparison of ddPCR and BC antibiotic guidance rate and effective rate.

Detection method	Adjusted cases (n)			Adjustment rate (%)	Effective rate (%)	Following BC adjustment (n)	Foregoing ddPCR adjustment (n)	P value
	Upgrades	Same level	Relegation					
ddPCR	36	6	3	24.59	60.13	0	-	0.613
BC	7	2	0	20.93	53.33	-	0	0.488

### 3.4 Relationship between ddPCR results and prognosis

(1) The correlation between the number of species(genera) and prognosis

This investigation examined the correlation between the maximum number of microbial species/genus detected through ddPCR and patient discharge outcomes. Analysis revealed statistically significant differences in microbial species/genus detection between patients with positive and negative outcomes (P < 0.001). The data fit the regression model well (Hosmer-Lemeshow test, P = 0.463). For each additional species/genus detected, the odds of negative discharge outcomes increased by a factor of 1.224 (**Table 4**).

**Table 4:** Logistic regression parameters of the relationship between number of species/gerera and prognosis.

B	SE	OR	P	95%CI
0.799	0.156	2.224	< 0.001	(1.270, 2.981)

(2) Correlation between ddPCR copies and prognosis

**Figure 7:**
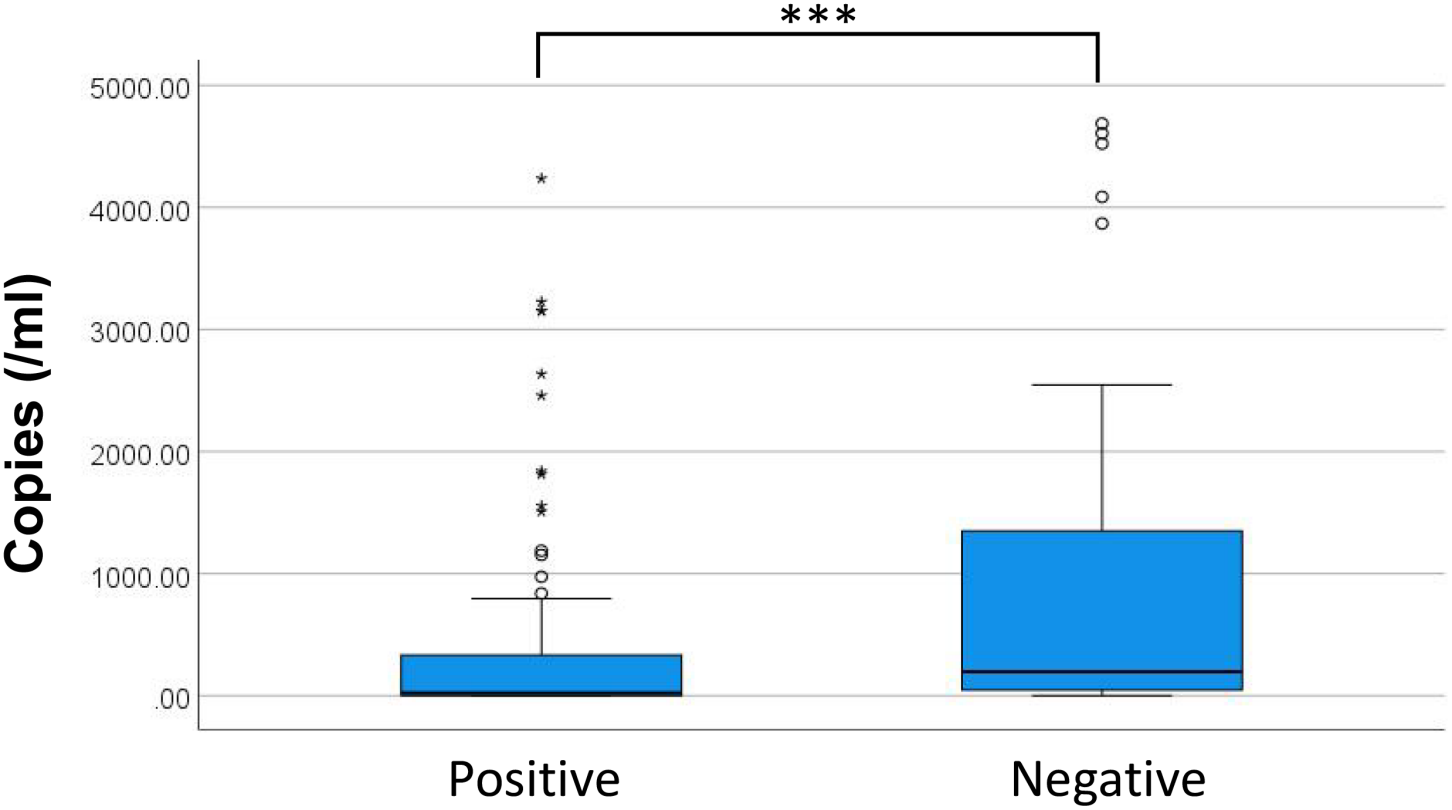
Distribution of copies measured in patients with different discharge outcomes. * discrete values; *** P<0.001.

To study examined the prognostic value of maximum microbial DNA copy numbers and by comparing levels among patients with different discharge outcomes. Analysis revealed significant differences in maximum copy numbers between positive and negative outcome groups (**Figure 7**, P < 0.001). While a positive correlation existed between higher copy numbers and negative discharge outcomes (P < 0.001, r_s_ = 0.326), this relationship did not fit a logistic regression model (Hosmer-Lemeshow test, P < 0.001). Evaluation of specific microbial genera showed no significant copy number differences between outcomes for either *Enterococcus* (P = 0.253) and Escherichia coli (P = 0.958), potentially attributable to limited sample sizes. While the predominant bacterial species during infection are identified based on maximum copy numbers, the presence of additional bacterial species may still impact clinical outcomes. Consequently, prognostic evaluation based exclusively on the copy number of the dominant species may not provide comprehensive clinical relevance.

(3) Relationship between dynamic changes in ddPCR results and prognosis

**Figure 8:**
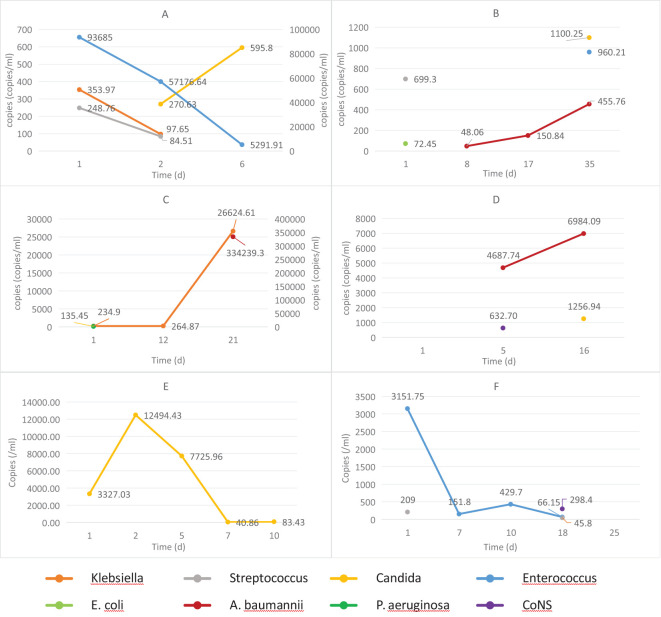
The dynamic changes in DNA loads among hospitalized patients with differing prognoses. **(A–D)** patients with negative outcomes; **(E, F)** patients with positive outcomes. *E. coli*, *Escherichia coli*; *P. aeruginosa*, *Pseudomonas aeruginosa*; *A. baumannii*, *Acinetobacter baumannii*; CoNS, Coagulase-negative Staphylococci.

Among BSI cases, 13 patients underwent ≥3 ddPCR follow-up tests. Exclusion criteria eliminated 7 cases due to strain variation, potential contamination, or other reasons. The remaining 6 patients qualified dynamic copy number analysis, comprising 4 cases with negative discharge outcomes and 2 with positive outcomes. **Figure 8** illustrates the temporal DNA load patterns in these patients. Negative outcomes cases demonstrated either progressive increases in major pathogen copy numbers or initial declines followed by new infections. Conversely, positive outcomes cases exhibited consistent decreases in major pathogen copy numbers without new infections.

### 3.5 The threshold to identify false positives

**Figure 9:**
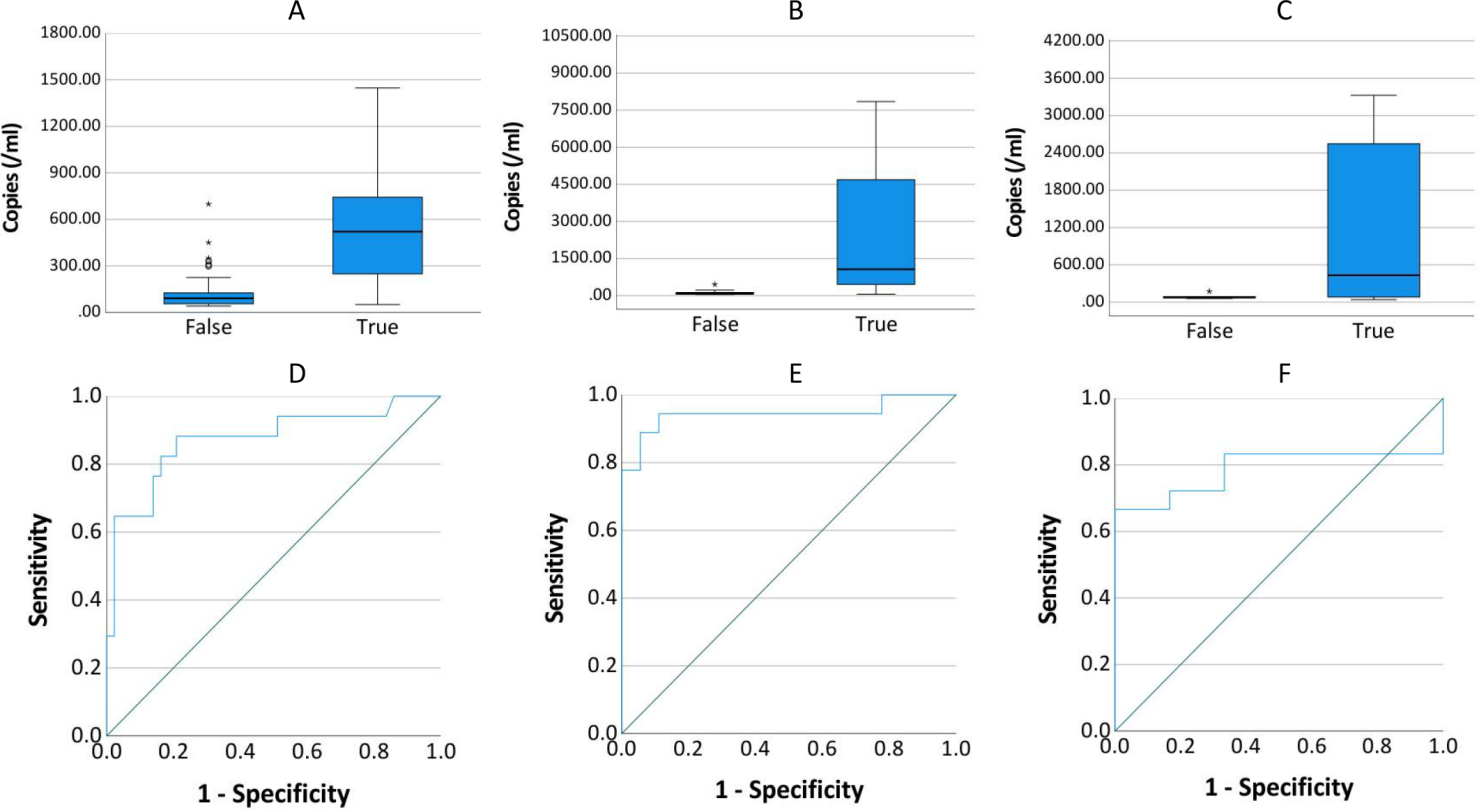
Copy number distribution and ROC curve by infection status. **(A–C)** show the distribution of copies in true-positive and false-positive results for Streptococcus or CoNS (n=60), A. baumannii (n=36), and Candida (n=24), respectively. **(D–F)** present the receiver operating characteristic (ROC) curves evaluating the diagnostic accuracy of copies for true-positive detection in each pathogen group.

All positive ddPCR results underwent clinical validation by two independent infectious disease specialists, incorporating evaluation of high-risk factors, clinical manifestations, laboratory tests, imaging examinations, reactions to antibiotics, and clinical outcomes. **Figure 9** presents the receiver operating characteristic (ROC) curves analyzing DNA copy number thresholds for distinguishing false positives among *Streptococci*, CoNS, *A. baumannii* complex, and *Candida*. Corresponding logistic regression parameters and comprehensive ROC curve metrics appear in **Tables 5** and **6** respectively.

**Table 5:** Diagnostic performance of ddPCR in identifying *Streptococcus*, CoNS, *A. baumannii* Complex, and *Candida* infections.

Species	B	SE	OR	P	OR 95%CI	Hosmer-Lemeshow, P
*Streptococcus* or CoNS copies/100*	0.704	0.198	2.021	< 0.001	(1.270, 2.981)	0.086
*A. Baumannii* copies/100*	1.026	0.430	2.791	0.017	(1.201, 6.487)	0.841
*Candida* copies/100*	0.781	0.786	2.184	0.320	(0.468, 10.188)	-

Spearman correlation analysis of Candida copies and pathogenic possibility: r_s_=0.431, P=0.036.

*Detected copies were scaled by a factor of 1/100 prior to logistic regression analysis.

**Table 6:** Diagnostic performance of ddPCR in identifying *Streptococcus*, CoNS, *A. baumannii* Complex, and *Candida* infections.

Bacterial Species	AUC	P	Detection threshold (copies/mL)	Jorden index	Sensitivity	Specificity
*Streptococcus* or CoNS	0.873	< 0.001	132.545	0.673	0.882	0.791
*A. baumannii*	0.944	< 0.001	182.70/262.24	0.833	0.944/0.889	0.889/0.944
*Candida*	0.787	0.039	174.775	0.667	0.667	1

The relationship between Candida copy numbers and true positive potential demonstrated discordant statistical patterns. Logistic regression modeling failed to establish significant association (P = 0.320). In contrast, Spearman correlation analysis revealed a statistical significantly positive correlation (r_s_ = 0.431, P = 0.036) between Candida DNA load and confirmed true positive status.

## 4 Discussion

This investigation evaluated the efficacy of ddPCR and BC for rapid pathogen identification, antimicrobial therapy guidance, and prognostic assessment in elderly BSI patients. ddPCR demonstrates superior resistance to blood-derived PCR inhibitors while maintaining high sensitivity and rapid turnaround time, exhibiting substantial diagnostic potential for pathogen detection (Chen et al., 2021). Although previous studies have validated its clinical utility (Ziegler et al., 2019; Hu et al., 2021; Shao et al., 2022; Wu et al., 2022; Lin et al., 2023; Liu et al., 2023; Li et al., 2024), limited data exist regarding application in elderly BSI populations, which exhibit distinct clinical manifestations, diagnostic challenges, and disease outcomes (Leibovici-Weissman et al., 2021). Considering the differential clinical presentation, diagnostic challenges, and prognosis of BSI in elderly patients compared to the general adult population, the enhanced sensitivity and specificity of ddPCR may provide particular diagnostic advantages for this vulnerable patient demographic.

Existing literature reported ddPCR positive rates of ranging from 41.1% to 83.3% in suspected BSI patients (Hu et al., 2021; Wu et al., 2022; Li et al., 2024). The current investigation demonstrated pathogen detection in 59% of elderly BSI patients using ddPCR versus pathogens 21% BC, with and combined methodology achieving 65% positivity. Analysis revealed 94.82% of all bloodborne pathogens identified fell within the ddPCR detection range, indicating that although the detection range of ddPCR remains limited, it covers the majority of clinically relevant microorganisms and satisfies routine diagnostic requirements.

BC, currently regarded as the diagnostic gold standard for BSI, demonstrates limited sensitivity (approximately 20%) (Hu et al., 2021; Sun et al., 2022) and requires prolonged turnaround time (1-3 days) (Ehren et al., 2020; Kim et al., 2021), creating substantial obstacles for rapid clinical diagnosis. This present study employed clinical assessment as the reference standard to evaluate the diagnostic efficiency of ddPCR and BC. Comparative analysis revealed markedly superior sensitivity for ddPCR (59.61% versus 20.57%). To mitigate potential subjective in the clinical interpretation, discordant results between ddPCR and BC were systematically analyzed using supplementary etiological evidence. This validation process showed enhanced ddPCR sensitivity (90.63%) and significantly higher PPV value compared to BC (**Table 2**). Despite its advantages, ddPCR exhibits detection limitations for specific pathogens including *Haemophilus influenzae* and *Bacteroides fragilis*. Expanding the detection range of ddPCR based on clinical epidemiology data could enhance its diagnostic utility. The median TAT for ddPCR and BC was 7.38 h and 19.60 h respectively, showing discrepancy with results of Yin et al (Yin et al., 2024). This discrepancy may be due to our study defining the BC TAT endpoint as the reporting of positive results rather than AST results.

This investigation further examined the distribution of ddPCR copy numbers across BC results. Quantitative analysis revealed significantly elevated ddPCR copy numbers in BC+ samples compared to BC-, though this relationship did not demonstrate adequate fit for logistic regression modeling. Limited sample size precluded detailed evaluation of species-specific ddPCR copy number correlations with BC outcomes. Notably, while ddPCR exhibited superior sensitivity to BC, discordant results were observed in 12 BC+/ddPCR- cases. Subsequent clinical evaluation confirmed 7 of these as true positives, all involving pathogens within the theoretical detection range of the ddPCR assay. As a retrospective study, this investigation included paired ddPCR and BC tests conducted within 24 hours but not necessarily simultaneously. Given the cyclical nature of pathogenemia (Forrester), sample collection timing significantly impacts detection sensitivity, with peak pathogenemia period yielding higher positivity rates compared to trough phases. Furthermore, analogous discrepancies have been documented in prospective studies employing simultaneous blood samples collection (Wu et al., 2022). These observations suggest potential technical influences, including variations in ddPCR testing or inherent instrument detection characteristics.

As the diagnostic gold standard for BSI, BC serves dual critical functions: establishing definitive etiological diagnosis and guiding antibiotic selection. Clinical evidence demonstrates the potential complementary role of ddPCR in antimicrobial stewardship. Lin et al. found that antibiotic regimen modifications in 51.2% of patients based on ddPCR results (Lin et al., 2023), while Li et al. reported observed inadequate empirical antimicrobial coverage relative to ddPCR results in 63.3% of patients (Li et al., 2024). These findings indicate ddPCR may provide valuable therapeutic guidance, particularly when BC results remain negative or inconclusive. In this study, the antibiotic adjustment rates for ddPCR and BC were 24.59% versus 20.93%, and effectiveness rates were 60.13% versus 53.33%, with no significant differences. Despite this, ddPCR’s higher sensitivity may offer better antibiotic guidance for elderly BSI patients and support earlier antibiotic adjustments. While antibiotic adjustments typically follow BC and AST, this study found ddPCR-based changes needed no later revision, and BC/AST-based changes were not influenced by prior ddPCR results. These findings suggest that while ddPCR, due to its higher sensitivity, demonstrates certain advantages in guiding antibiotic use, BC combined with AST remains an important basis for antibiotic decision-making.

Previous investigations by Shao, Ziegler, et al. established significant associations between elevated microbial DNA loads in BSI patients and three clinical parameters: increased inflammation, more severe disease, and worse prognosis (Ziegler et al., 2019; Shao et al., 2022). The current findings corroborate these observations, demonstrating statistically significant differences in both maximum copy numbers and more species/genera than those with favorable outcomes. During early in BSI, reduced pathogen loads correlate with both diminished BC positive rates and absence of organ dysfunction. Timely antibiotic use based on ddPCR can prevent severe complications and improve prognosis. During disease progression, increasing microbial loads correlate with enhanced BC positive rates, yet may coincide with missed therapeutic windows, leading to suboptimal treatment responses and unfavorable clinical outcomes. Notably, while significant differences in pathogen copy numbers exist between patient groups, absolute quantitative values demonstrate limited predictive capacity for individual prognosis, a finding consistent with previous reports by Zhao, Shao, et al (Shao et al., 2022; Zhao et al., 2024). The dynamic changes of microbial DNA copy number may provide valuable prognostic supplementation. However, the limited sample size of current investigation for serial monitoring necessitates further validation studies to establish the clinical utility of copy number kinetics in prognosis prediction.

The present study addressed the critical diagnostic challenge of differentiating true positives from false positives (including contamination and transient bacteremia/fungemia) in clinical microbiological testing, with particular focus on *Streptococcus* or CoNS, *A. baumannii*, and *Candida*. These organisms can asymptomatically colonize patients but also cause infections in immunocompromised individuals (Beigi et al., 2004; Bougnoux et al., 2006; Grice et al., 2009; Knauf et al., 2022; Lopes and Lionakis, 2022). Comparative analysis revealed that optimal diagnostic performance for *Streptococcus* & CoNS, *A. baumannii* complex, and Candida species required elevated threshold values (132.545, 182.70/262.24, and 174.775 copies/mL, respectively) compared to the overall diagnostic threshold of 45 copies/mL. At these optimal thresholds, the sensitivity and specificity were: 0.882 versus 0.791, 0.944 versus 0.889 (0.0.889 versus 0.944), 0.667 versus 1. The current threshold analysis presents two notable limitations: the restricted sample size and potential supervisor bias in clinical determination of true and false positive results. These constraints necessitate further investigation through larger-scale studies employing more rigorous, predefined clinical criteria to validate the present findings.

This study demonstrates that ddPCR, as an emerging pathogen detection methodology, provides superior sensitivity and rapid turnaround time, facilitating early diagnosis and therapeutic intervention for BSI in elderly populations, ultimately leading to improved clinical outcomes. While the inherent limitations of PCR technology constrain its detectable spectrum, this approach nevertheless encompasses the predominant clinically relevant microorganisms responsible for BSI. This investigation represents the first comprehensive comparison establishing advantages of ddPCR over BC in elderly BSI patients, while proposing that early implementation of ddPCR testing may confer prognostic benefits. Furthermore, the study systematically assesses ddPCR performance for microorganisms with high false positives potential and establishes novel diagnostic thresholds to enhance clinical interpreting of results.

The current investigation faces constraints in evaluating the diagnostic and prognostic utility of ddPCR value at the species/genus levels due to limited sample size. The retrospective design precluded simultaneous collection of ddPCR and BC samples, with potential confounding variables beyond methodological differences potentially contributing to observed result variations. These limitations necessitate prospective validation studies to confirm these findings. For clinical relevance, the study employed physician-determined clinical assessment as the reference standard for BSI diagnosis and discrimination between true and false positives results. Despite employing independent case assessments by multiple experienced clinicians to ensure reproducibility, inherent subjective in clinical diagnostic process remains unavoidable. This methodological limitation may lead to partial overestimation of the diagnostic advantages attributed to ddPCR. Consequently, the reported diagnostic superiority of ddPCR requires additional verification through rigorous studies. Future investigations should incorporate predefined composite reference standards in accordance with contemporary best practices for diagnostic accuracy research.

## 5 Conclusion

BSI in elderly patients frequently manifests with nonspecific clinical presentations, while collected specimens demonstrate increased susceptibility to contamination. The enhanced analytical sensitivity of ddPCR relative to conventional BC confers significant advantages for the early BSI detection in this demographic. Nevertheless, positive results from both ddPCR and BC require careful interpretation alongside clinical presentation and supplementary diagnostic evaluations. Implementation of composite clinical adjudication incorporating predefined criteria represents a critical methodological advancement aligned with contemporary diagnostic standards. Further refinement of pathogen-specific detection thresholds, particularly for microorganisms associated with contamination or transient bacteremia, would improve the reliability of positive results.

## Data Availability

The raw data supporting the conclusions of this article will be made available by the authors, without undue reservation.
