# Supplementary material for: ddPCR Enhances early diagnosis, treatment, prognosis, and pathogen verification in elderly BSI
Source: Front Cell Infect Microbiol. 2025 Jul 10;15:1605795. doi: 10.3389/fcimb.2025.1605795 (PMC12286947; doi:10.3389/fcimb.2025.1605795)
Supplement: Supplementary file 1 [file Table1.docx]

Supplemental table 1 Inclusion and exclusion criteria

| Inclusion Criteria |
| --- |
| 1. 1. Age ≥60 years old; 2. Elderly inpatients suspected of bloodstream infection (BSI) must fulfill at least three of the following diagnostic criteria:    1. Meet ≥2 SIRS diagnostic criteria:       1. Body temperature >38℃ or <36℃       2. Heart rate > 90 beats/min       3. Respiratory rate > 20 breaths/min or PaCO2 < 32mmHg       4. White blood cell count >12×10^9/L or <4×10^9/L or immature granulocyte >10%    2. Experience a consistent or sudden significant increase in N%/CRP.    3. Show a consistent or sudden significant increase in PCT/G/GM.    4. Exhibit other positive evidence of bloodstream infection within 3 days.    5. Have an infection at other sites, such as the respiratory tract, urinary tract, digestive system, abdominal cavity, skin, or other locations.    6. Present with other relevant risk factors.       1. Immune deficiency: glucocorticoids, the use of chemotherapy drugs, the body's immune function test results are obviously abnormal, immunosuppressive complications (tumor, hematological disease, liver and kidney failure, AIDS, splenectomy, diabetes, obesity or sarcopenia);       2. Previous diagnosis of immune deficiency or family history of immune deficiency;       3. The length of stay in the hospital ≥14 days or stay in the ICU≥3 days;       4. Invasive medical procedures, such as mechanical ventilation, arterial and venous catheter placement.       5. History of major thoracic and abdominal surgery within 7 days. |
| Exclusion Criteria |
| 1. No ddPCR examination was performed; 2. The medical record is incomplete. |

Supplemental table 2 Reaction solution and fluorescent channel detection targets

| Panel | FAM | VIC | ROX | CY5 | CY5.5 |
| --- | --- | --- | --- | --- | --- |
| 01 | Pseudomonas aeruginosa | Escherichia coli | KleBSIella pneumoniae | Acinetobacter baumannii | / |
| 02 | Staphylococcus aureus | Candida spp. | Enterococcus spp. | Streptococcus spp. | / |
| 03 | Stenotrophomonas maltophilia | Enterobacter cloacae | Proteus mirabilis | Coagulase-negative staphylococci | Serratia marcescen |
| 06 | KPC | mecA | OXA-48 | NDM/IMP | vanA/vanB |

Supplemental table 3 Classification of antibiotics

| **classification** | **1** | **2** | **3** |
| --- | --- | --- | --- |
| Anti-bacterial medications | penicillins^1^ | Third and fourth generation cephalosporins^3^ | Penicillin/cephalosporin compound preparations (beta -  Lactamase inhibitors)^5^ |
|  | First and second generation cephalosporin^2^ | Fluoroquinolones^4^ | Tigecycline |
|  | Compound sulfamethoxazole | Ornidazole | Linezolid |
|  | Erythromycin | Azithromycin | Carbapenems and their compound preparations^6^ |
|  | Amikacin | Minocycline | Glycopeptides^7^ |
|  | Doxycycline | Clindamycin | Polymyxin B |
|  | Metronidazole |  | Aztreonam |
| Anti-fungal medication | Fluconazole | Itraconazole, Voriconazole, carpofungin, amphotericin B |  |

^1^Penicillins: including penicillin, oxacillin, amoxicillin; ^2^The first and second generation of cefuroxime: including cefuroxime (ester), cefaclor; ^3^The third and fourth generation of ceftriaxone and cephalosporin: including ceftriaxone, cefotaxime, ceftazidime, cefoperazone, cefixime, cefdini, cefzoxime, cefoxime, cefoxitin;  ^4^Fluoroquinolones: including levofloxacin, moxifloxacin; ^5^Penicillin/cephalosporin compound preparations (β-lactamase inhibitors) : piperacillin/tazobactam, cefoperazone/sulbactam; ^6^Carbapenems and their compound preparations: imipenem/Cilastatin, meropenem; ^7^Glycopeptides: Vancomycin, Teicoplanin

**The prompts provided to the Generative AI**: You are now a professional academic touch-up specialist. Please polish the English draft I am sending you next. After analyzing the paragraph, give suggestions for polishing in terms of sentence structure, grammar, diction, clarity of expression, etc., while maintaining the original meaning. Directly output the paragraph after polishing. In addition, please provide a table describing the original sentence, the revised sentence, and the reason for the revision, in which the third column of the reason for the revision describes what vocabulary or grammatical changes have been made(only this column is explained in Chinese).
